# Management of ST-segment elevation myocardial infarction in comparison to European society of cardiology guidelines in Alexandria University Hospitals, Egypt

**DOI:** 10.1186/s43044-023-00332-x

**Published:** 2023-01-21

**Authors:** Amr Kamal, Amr Zaki, Ahmed Abdelaaty, Moustafa Madkour

**Affiliations:** Cardiology and Angiology Department, Faculty of Medicine, Alexandria University, Champollion Street, Azareeta, Alexandria Egypt

**Keywords:** Primary percutaneous coronary intervention, Reperfusion therapy, ST elevation myocardial infarction, Thrombolytic therapy

## Abstract

**Background:**

For patients with ST-elevation myocardial infarction (STEMI), early reperfusion with primary percutaneous coronary intervention (PPCI) or thrombolytic treatment is essential to prevent major adverse cardiac events. The aim of the study is to compare the current status of managing STEMI patients at **** with European Society of Cardiology guidelines recommendations. Prospective cohort of all patients presenting with ST-elevation myocardial infarction (STEMI) between March 2020 and February 2021 in Alexandria University hospitals. Reporting patterns, causes of delay, and reperfusion status for all STEMI patients were noted. MACE: (Mortality, Re-infarction, Stroke, or Heart failure) was reported and compared among different management strategies.

**Results:**

The study was conducted over one year on 436 patients, 280 (64.2%) of them underwent PPCI, 32 (7.3%) received thrombolysis, and 124 (28.5%) had a conservative strategy. Patients’ mean age was 55.2 years, 72.2% were smokers and 80.9% were men. Family history was positive in 14.2% of patients, 33.5% had diabetes, 7.3% had renal impairment, and 41.5% had hypertension. The median pre-hospital waiting time was 360 min; the mean pre-hospital waiting time was 629.0 ± 796.7 min. The median Emergency Room waiting time was 48.24 ± 89.30 min. The median time from CCU admission to wire crossing was 40.0 min with a mean value 53.86 ± 49.0 min. The mean ischemia duration was 408 min, while the total ischemic time was 372 min. All patients who presented within 12 h received reperfusion therapy either a PPCI or thrombolysis at a rate of 71.5%, with 35.0% of those patients achieving prompt reperfusion in accordance with ESC guidelines. The PPCI group mortality rate was 2.9%, in comparison to 12.9% in the conservative group, which was statistically significant (*P* < 0.001). Overall in-hospital mortality was 5.5%, and total MACE was 27.3%. A statistically significant difference was observed between the three management groups as regards MACE rate, being 15%, 28.1%, and 54.8% in PPCI, thrombolysis, and conservative groups, respectively.

**Conclusions:**

Despite financial and technical constraints, appropriate, timely reperfusion was near to achieving the ESC guidelines for the management of STEMI. The most common reperfusion strategy was PPCI, with an in-hospital death rate of less than 5% in the PPCI group. There was a concern about the increase in the total ischemia time due to some financial and technical constraints.

## Background

An ST-elevation myocardial infarction (STEMI) is a major cause of morbidity and mortality worldwide [1]. The acute therapy of STEMI focuses on the recanalization of the occluded coronary artery to provide immediate efficient reperfusion of the myocardium. Primary PCI, as opposed to fibrinolysis, has proved to improve outcomes in STEMI patients when treated within 120 min of diagnosis and has thus become the preferred reperfusion technique [2, 3]. STEMI patients referred to or presented to the Emergency room (ER) experience a significant and preventable PPCI-related delay due to many causes. First, ER routines and paperwork consume a major preventable delay. Second, missed diagnosis by under-trained personnel due to miss interpretation of patient symptoms or miss interpretation of electrocardiogram (ECG). Third, delay in performing ECG due to system overload. Finally, transferring the patient from ER to Critical Care Unit (CCU) or cath-lab plays a role in overall delay [4]. Ongoing enhancement of interventions and policies implemented in the past few decades have led to improvement in diagnosis. International recommendations urge conventional, research-based management. However, significant variance in the clinical results and management of STEMI published in various countries suggests inadequate implementation [5]. In order to ensure better implementation of guidelines and provide a higher quality of care, It is recommended to develop measurable quality indicators and conduct periodic audits to ensure the best possible care [6]. Our study focuses on reporting and monitoring the implementation of the 2017 European Society of Cardiology (ESC) Guidelines for STEMI management in our center.

## Methods

### Population study

A total of 444 patients were admitted to Alexandria University Hospitals Cardiology department between March 2020 and February 2021 with STEMI. Patients were treated either conservatively (*n* = 124), by thrombolysis (*n* = 32), or by Primary Percutaneous Coronary Intervention (PPCI) (*n* = 288). Eight patients from the PPCI group were excluded due to the presence of pre-specified exclusion criteria. Admission within 12 h of the onset of chest discomfort qualified patients for inclusion, while those who were admitted later (with no ongoing pain) were excluded from the reperfusion strategy. Reporting patterns, causes of delay, and reperfusion status for all STEMI patients were noted. MACE events (Mortality, Re-infarction, Stroke, or Heart failure) were reported and compared among different management strategies.

### Interventional procedures and adjunctive medications

All patients undergoing primary PCI were given 300 mg acetylsalicylic acid (ASA) and Clopidogrel (loading dose of 600 mg) or Ticagrelor (loading dose of 180 mg) together with high-intensity statins and anticoagulation (intravenous unfractionated heparin 70–100 IU/kg) regularly. Radial or femoral access was used to perform PPCI. The culprit lesion was bridged with a guide wire, and the infarct-related artery was engaged with an adequately sized guiding catheter. Standard procedures were used to insert stents or dilate balloons. All patients received drug-eluting stents (DES). PPCI was restricted to Infarct Related Artery (IRA) in the event of multi-vessel illness. The TIMI flow grade was reported at baseline and following the procedure.

### Data collection

Detailed history and physical examination were applied to all subjects, time variables and delays either pre-hospital, ER or CCU were documented. Intra-procedural and post-procedural complications were also reported. Follow up within hospital stay to detect any in-hospital events (mortality, re-infarction, stroke, bleeding complication, arrhythmias, heart failure).

### Statistical analysis

With the aid of the IBM SPSS software package version 20.0, data were uploaded into the computer and evaluated. (Armonk, NY: IBM Corp). Numbers and percentages were used to represent qualitative data. The normality of the distribution was examined using the Kolmogorov–Smirnov test. Interquartile range (IQR), mean, standard deviation, median, and range (minimum and maximum) were used to characterize quantitative data. At a 5% significance level, the obtained results were considered significant.

## Results

Baseline characteristics were summarized in Table 1, Age ranged from 27.0 to 89.0 years with mean value 55.23 ± 10.41 years. The majority of studied sample were male (80.9%) while (19.1%) were females. Smoking was the most prevalent cardiac risk factor, which was detected in 72.2% of the patients. There were 181 (41.5%) patients with hypertension and 146 (33.5%) individuals with diabetes mellitus.

**Table 1:** Demographic and clinical data (*N* = 436)

Risk factor	No	%	Mean ± SD
Age			55.23 ± 10.41
Male	353	80.9	
Female	83	19.1	
Smoker	315	72.2	
Cannabis	61	14	
Hypertension	181	41.5	
Diabetic	146	33.5	
Hyperlipidemia	134	30.7	
History of Acute Coronary Syndrome (ACS)	51	11.7	
History of PCI	23	5.3	
History of stroke	14	3.2	
History of CABG	2	0.5	
Family history of ACS	62	14.2	
CKD	32	7.3	
Killip class			
I	289	66.3	
II	118	27.1	
III	26	6	
IV	3	0.7	

According to the management strategy, subjects were distributed into three groups; PPCI group, the thrombolytic group, and the conservative group. Table 2 represents the number of subjects in each group and the reasons for not choosing PPCI as a strategy of choice. Two hundred and eighty patients (64.2%) underwent PPCI, thirty-two patients (7.3%) had thrombolytic therapy and 124 patients (28.4%) had a conservative strategy.

**Table 2:** Management strategy and cause

Type of management	No	%	Mean ± SD
PCI	280	64.2	
PPCI	276	63.3	
Rescue PCI	4	0.9	
Thrombolytic	32	7.3	
Operator not available	12	37.5	
Cath lab malfunction	20	62.5	
FMC to wire crossing(min)			48.13 ± 14.85
Conservative	124	28.4	
Evolved	103	83.0	
Arrested before PCI	2	1.6	
Cath lab malfunction			
Not candidate for thrombolytic	10	8.1	
Refusal of thrombolytic	3	2.4	
CI to thrombolytic	6	4.8	

As mentioned in Table 3 the mean pre-hospital delay was 629.0 ± 796.7 min (10.4 h), while the median was 360 min; 45.6% of cases were primarily caused by a delay in seeking medical attention.

**Table 3:** Time variables

	Mean ± SD	Median (IQR)
Pre-hospital delay (min) (*N* = 436)	629.0 ± 796.7	360.0 (240.0–600.0)
ER delay (min) (*N* = 436)	48.24 ± 89.30	40.0 (30.0–52.50)
CCU delay	53.86 ± 49.0	40.0 (30.0–60.0)
Door to crossing time (*N* = 280)	92.86 ± 54.66	70.0 (60.0–110.0)
Total ischemic time (min.) (280)	409.71 ± 176.80	409.71 ± 176.80
Door to crossing (min) (*N* = 280)	No	%
≤ 60	98	35
60–90	100	35.7
91–120	32	11.4
> 120	50	17.9
Cause of pre-hospital delay (*N* = 436)		
No delay (< 60)	3	0.7
Distance	73	16.7
Missed diagnosis	78	17.9
Delay seeking medical care	199	45.6
Refereed from other hospital	83	19
Cause of ER delay (*N* = 436)		
No Delay (< 15 min)	7	1.6
Missed diagnosis	44	10.1
Transfer delay	268	61.5
Diagnosis delay	117	26.8
Door to crossing time	92.86 ± 54.66	

In contrast, the average ER delay time was 48.24 ± 89.30 min. With a mean value of 53.86 ± 49.0 min, the median time from CCU admission to wire crossing was 40.0 min. The mean ischemia duration was 408 min (6.8 h), while the overall ischemic time was 372 min (6.2 h). All STEMI patients who presented within 12 h received reperfusion therapy (PPCI or thrombolysis) at a rate of 71.5 percent, with 35.0% achieving prompt reperfusion in accordance with ESC guidelines for the management of STEMI (Tables 4, 5).

**Table 4:** Comparison between the different studied types of management according to risk factors and Killip Class on presentation

Risk factor	Total (*n* = 436)		PCI (*n* = 280)		Thrombolytic (*n* = 32)		Conservative (*n* = 124)	
	No	%	No	%	No	%	No	%
Age; mean ± SD	55.23 ± 10.41	54.73 ± 10.27	52.06 ± 8.72	57.18 ± 10.84				
Male	353	80.9	226	80.7	29	90.6	98	79.0
Female	83	19.1	54	19.3	3	9.4	26	21.0
Smoker	315	72.2	201	71.8	29	90.6	85	68.5
Cannabis	61	14	38	13.6	5	15.6	18	14.5
Hypertension	181	41.5	115	41.1	8	25.0	58	46.8
Diabetic	146	33.5	87	31.1	14	43.8	45	36.3
Hyperlipidemia	209	47.9	127	45.4	16	50.0	66	53.2
History of acute coronary syndrome (ACS)	51	11.7	31	11.1	4	12.5	16	12.9
History of PCI	23	5.3	17	6.1	1	3.1	5	4.0
History of stroke	14	3.2	7	2.5	0	0.0	7	5.6
History of CABG	2	0.5	2	0.7	0	0.0	0	0.0
Family history of ACS	62	14.2	34	12.1	9	28.1	19	15.3
CKD	32	7.3	17	6.1	2	6.3	13	10.5
Killip class								
I	289	66.3	221	78.9	22	68.8	46	37.1
II	118	27.1	56	20.0	6	18.8	56	45.2
III	26	6	3	1.1	4	12.5	19	15.3
IV	3	0.7	0	0.0	0	0.0	3	2.4

**Table 5:** Comparison between the different studied types of management according to different pre and in-hospital delay times

	Total (*n* = 436)	PCI (*n* = 280)	Thrombolytic (*n* = 32)	Conservative (*n* = 124)
Pre-hospital delay (min)				
Mean ± SD	629.0 ± 796.7	316.9 ± 167.6	291.9 ± 126.7	1420.9 ± 1137.3
Median (IQR)	360 (240–600)	290 (180–420)	240 (195–360)	920 (570–1800)
ER delay (min)				
Mean ± SD	48.24 ± 89.30	39 ± 23.49	27.81 ± 10.16	74.38 ± 161.0
Median (IQR)	40 (30–52.5)	35 (30–45)	30 (20–30)	59 (40–60)
CCU delay				
Mean ± SD		53.86 ± 49	–	–
Median (IQR)		40 (30–60)	–	–
Pre-hospital delay (min)				
Mean ± SD	629.0 ± 796.7	316.9 ± 167.6	291.9 ± 126.7	1420.9 ± 1137.3
Median (IQR)	360 (240–600)	290 (180–420)	240 (195–360)	920 (570–1800)
ER delay (min)				
Mean ± SD	48.24 ± 89.30	39 ± 23.49	27.81 ± 10.16	74.38 ± 161.0
Median (IQR)	40 (30–52.5)	35 (30–45)	30 (20–30)	59 (40–60)
CCU delay				
Mean ± SD		53.86 ± 49	–	–
Median (IQR)		40 (30–60)	–	–

Tables 4 and 5 compare different types of management studied in this research based on risk factors and Killip Class on presentation and pre- and in-hospital delay times, respectively

**Fig. 1:**
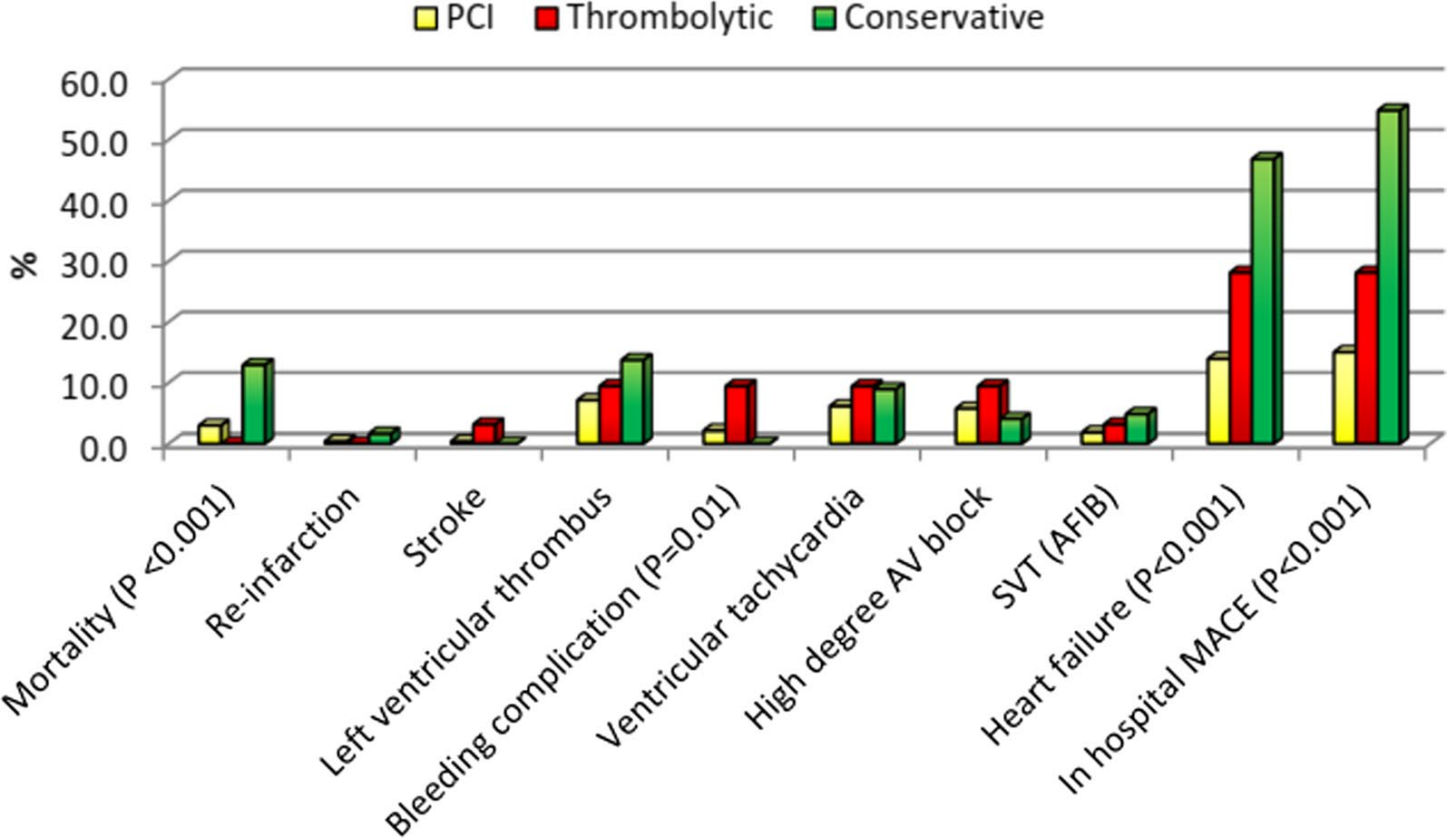
Comparison between the different studied types of management according to hospital events (*n* = 436)

Figure 1 and Table 6 compare the various examined care options according to hospital events and reveals statistically significant differences in favor of the PPCI group for mortality (*P* < 0.001), bleeding complications (*P* = 0.010), heart failure (*P* < 0.001) and in hospital Major adverse cardiac events (MACE) (*P* < 0.001).

**Table 6:** Comparison between the different studied types of management according to hospital events

Hospital events	Total (*n* = 436)		Type of management						χ^2^	*P*
			PCI (*n* = 280)		Thrombolytic (*n* = 32)		Conservative (*n* = 124)			
	No	%	No	%	No	%	No	%		
Mortality	24	5.5	8	2.9	0	0.0	16	12.9	18.686	< 0.001^*^
Re-infarction	3	0.7	1	0.4	0	0.0	2	1.6	2.173	^MC^*P* = 0.375
Stroke	2	0.5	1	0.4	1	3.1	0	0.0	3.978	^MC^*P* = 0.216
Left ventricular thrombus	40	9.2	20	7.1	3	9.4	17	13.7	4.449	0.108
Bleeding complication	9	2.1	6	2.1	3	9.4	0	0.0	8.542^*^	^MC^*P* = 0.010^*^
Ventricular tachycardia	31	7.1	17	6.1	3	9.4	11	8.9	1.288	0.525
High degree AV block	24	5.5	16	5.7	3	9.4	5	4.0	1.462	0.481
SVT (AFIB)	12	2.8	5	1.8	1	3.1	6	4.8	3.234	^MC^*P* = 0.160
Heart failure	106	24.3	39	13.9	9	28.1	58	46.8	50.658^*^	< 0.001^*^
In hospital MACE^#^	119	27.3	42	15.0	9	28.1	68	54.8	68.747^*^	< 0.001^*^

^*^Statistically significant at *P* ≤ 0.05

^#^All variables with *P* < 0.05 was included in the multivariate

Regression analysis for different parameters affecting in-hospital MACE is depicted below (Table 7).

**Table 7:** Univariate and multivariate logistic regression analysis for the parameters affecting in-hospital MACE (*n* = 119 vs. 317)

	In hospital MACE		Univariate		Multivariate	
	No® (*n* = 317)	Yes (*n* = 119)	OR (LL–UL 95%C.I)	*P*	OR (LL–UL 95%C.I)	*P*
Type of management						
PCI	238 (75.1%)	42 (35.3%)	0.181 (0.115–0.285)	< 0.001^*^	0.337 (0.136–0.832)	0.018^*^
Thrombolytic	23 (7.3%)	9 (7.6%)	1.046 (0.469–2.330)	0.913		
Conservative	56 (17.7%)	68 (57.1%)	6.214 (3.908–9.882)	< 0.001^*^	2.549 (1.014–6.407)	0.047^*^
Age (years)	54.58 ± 10.0	56.97 ± 11.29	1.023 (1.002–1.044)	0.034^*^	1.016 (0.992–1.040)	0.195
Hypertension	125 (39.4%)	56 (47.1%)	1.365 (0.893–2.088)	0.151		
Diabetic	99 (31.2%)	47 (39.5%)	1.437 (0.928–2.227)	0.104		
Smoker	235 (74.1%)	80 (67.2%)	0.716 (0.453–1.131)	0.152		
Type of STEMI						
Extensive anterior	32 (10.1%)	25 (21.0%)	2.369 (1.336–4.200)	0.003^*^	19.701 (3.467–111.94)	0.001^*^
Anterior	134 (42.3%)	73 (61.3%)	2.167 (1.408–3.335)	< 0.001^*^	9.844 (1.941–49.911)	0.006^*^
Inferior	137 (43.2%)	22 (18.5%)	0.298 (0.178–0.498)	< 0.001^*^	2.479 (0.523–11.743)	0.253
Lateral	20 (6.3%)	2 (1.7%)	0.254 (0.058–1.103)	0.067		
Posterior	51 (16.1%)	8 (6.7%)	0.376 (0.173–0.818)	0.014^*^	1.329 (0.488–3.618)	0.578

*OR* Odd`s ratio, *C.I* Confidence interval, *LL* lower limit, *UL* upper limit

^*^Statistically significant at *P* ≤ 0.05

^#^All variables with *P* < 0.05 were included in the multivariate analysis

## Discussion

Healthcare system performance, as well as patient education and behavior, are the cornerstone in the management of STEMI and improving clinical outcomes. The emerging need to conduct this study is clear with the lack of a STEMI network in Alexandria. Reperfusion delays are the most easily audited index in STEMI management of quality care. A patient’s delay or a healthcare system’s delay is what delays the reperfusion strategy. Delay in the healthcare system is the period between FMC and reperfusion. A delay in the healthcare system can occur at many stages: Emergency Medical Services (EMS) delay, ER delay and CCU delay.

As regarding baseline characteristics, Zeymer et al. [7] described reperfusion strategy and in-hospital outcomes for STEMI patients based on 11,462 patients in Association for Acute Cardiovascular Care (ACVC)- European Association of Percutaneous Coronary Intervention (EAPCI) EurObservational programme (EORP) STEMI registry. The mean age was 61.0 year. The majority were males (76.9%), smoking and diabetes were less in percentage than our study 45.7% and 26.7%, respectively, while hypertension (47.9%), hyperlipidemia (38.5%) were higher in percentage than in our study. In a meta-analysis studying STEMI epidemiology, management, and outcomes in five Asian-Pacific countries, twenty studies, including 158 420 patients, were under investigation. Tern et al. [8] stated that 78.7% of them were males, 30.5% were diabetic, 36.7% had Hyperlipidemia. Those results are similar to demographic data in our study except for the mean age of STEMI patients that was higher (61.6 years), hypertension as risk factor was higher (53.7%) and smoking was less (53.0%) (Table 4).

The mean value of pre-hospital delay in our study was 629.0 ± 796.7 min (10.4 h) (Table 5). The main cause of Pre-hospital delay was a delay in seeking medical care (45.6%), which indicates the poor application of medical education to the general population. 17.9% of the patients had been missed diagnosed, which on the other hand, indicates deficient medical training for ER physicians and General practitioners. Lack of PCI capable facilities led to long distance and difficult transportation for 16.7% of the patients. Zeymer et al. [7] reported that average time from symptoms onset to first medical contact was 221.6 ± 460.6 min which is significantly lower than in our study. Shaheen et al. [9] studied the current practice of STEMI management in Egypt and reported that delay in seeking medical advice is the main cause of pre-hospital delay and 24% of patients presenting to PPCI hospitals arrive to the hospital within 2 h of chest pain which is significantly higher than in our study.

ER delay was appointed as the time from ER admission to CCU admission. Upon analysis, the main factor of delay was transfer delay (61.5%) due to deficient numbers of transporting equipment and personnel. ER high volume admissions with the lack of ER beds and equipment also play an important role as it delays reaching to diagnosis. Steg et al. [10] studied a total of 1204 patients, 33.1% of them were taken to the ER before being admitted to the CCU, whereas 66.9% were admitted immediately to the CCU laboratory. Direct transfer to the CCU was linked to a quicker time between the onset of symptoms and admission to the CCU (244 *vs*. 292 min; *P* < 0.001) and a higher reperfusion rate (61.7% *vs*. 53.1%; *P* = 0.001). Choosing not to use the ER also decreased five-day mortality rates (4.9% v 8.6%; *P* = 0.01).

As the primary PCI center, Door to balloon was calculated from the first medical contact in our emergency department through CCU to Cath lab. Mean door to crossing time was 92.86 ± 54.66 min, and the median time was 70.0 (60.0–110.0). ESC latest guidelines for the management of STEMI described Timely PPCI in PPCI capable hospital as less than 60 min from door to balloon, Zeymer et al. [7] reported 54.4% of the studied population had timely reperfusion, while in our center as PPCI capable center had 35% timely reperfusion. Tern et al. [8] stated that the median door to balloon time was 63.5 (39.7–87.2), which was consistent with our study.

In-hospital mortality, in our study was 5.5% (*n* = 436) irrespective of the type of management, while in the PPCI group, 2.9% (*n* = 280), and 12.9% (*n* = 124) in the conservative management group, with a statistically significant difference (*P* < 0.001). Within one-month mortality rate was 3.4% irrespective of the type of management, with the highest in the conservative group, 8.2%, with a statistically significant difference from other groups (MCp = 0.001). MACE rate was 27.3% (*n* = 436) irrespective of the type of management, while the MACE rate was 15% in the PPCI group, 28.1% in the thrombolytic group, and 54.8 in the conservative group with a statistically significant difference (*P* < 0.001). One month follow-up MACE rate was 17.9% irrespective of the type of management, with the highest in the conservative group at 34.1%, with a statistically significant difference from other groups (*P* = 0.001) (Table 6). Song et al. [11] reported an in-hospital mortality rate in primary PCI-treated patients of 3.2%, a heart failure rate of 11.3%, and MACE rate of 16.9%, which is consistent with our study in mortality but less in heart failure rate and higher in MACE rate. Zeymer et al. [7] reported in-hospital mortality of 4.4% (*n* = 11,462) irrespective of the type of management, while mortality occurred in 3.1% of the PPCI group (*n* = 8275), 4.4% (*n* = 2160) in thrombolytic group and 14.1% (*n* = 1027) conservative management group, which is consistent with our study.

In-hospital mortality was observed by Shaheen et al. [12] to be 4.65% in Egypt, 2.10% in primary PCI, 4.97% in thrombolysis, and 18.87% in no-reperfusion patients, which was higher than our study in no-reperfusion group.

## Conclusions

Despite financial and technical constraints, timely reperfusion was near to achieving the ESC guidelines for the management of STEMI. The most common reperfusion strategy was PPCI, with an overall in-hospital death rate of less than 5%. There was a concern about the increase in the total ischemia time due to some financial and technical constraints.

This study emphasized the essential need for the expansion of public awareness and patient education and sufficient training of general practitioners and ER physicians to improve STEMI management. Design and application of the STEMI network while improving the EMS performance in **** are a must.

## Data Availability

The dataset used during the current study is available from the corresponding author on reasonable request.

## Abbreviations

STEMI: ST-elevation myocardial infarction
PCI: Percutaneous coronary intervention
MACE: Major adverse cardiac events
ESC: European Society of Cardiology
PPCI: Primary percutaneous coronary intervention
ER: Emergency room
CCU: Critical care unit
ECG: Electrocardiogram
DES: Drug-eluting stents
IRA: Infarct-related artery
ACS: Acute coronary syndrome
EMS: Emergency medical services
